# Unilateral Renal Tumor Cryoablation and Contralateral Radical Nephrectomy of Bilateral Renal Tumors by Transumbilical 3D Multichannel Laparoendoscopic Single-Site Surgery

**DOI:** 10.1089/cren.2017.0120

**Published:** 2018-04-01

**Authors:** Guopeng Yu, Ke Zhang, Wenzhi Li, Long Li, Yanliang Zhao, Yiwei Wang, Mingxi Xu, Jun Da, Guoqin Dong, Zhong Wang, Bin Xu

**Affiliations:** ^1^ Department of Urology, Shanghai Ninth People's Hospital, Shanghai Jiao Tong University School of Medicine, Shanghai, China.; ^2^ Department of Urology, The People's Hospital of Xiangyun, Dali Bai Autonomous Prefecture, Yunnan, China.

**Keywords:** transumbilical, LESS, cryoablation, bilateral kidney tumors

## Abstract

***Background:*** Unilateral renal tumor cryoablation and contralateral radical nephrectomy of bilateral renal tumors were performed by transumbilical three-dimensional (3D) multichannel laparoendoscopic single-site (LESS) surgery, in an attempt to verify the feasibility and safety of the procedure, sum up the operational experience, and evaluate the surgical outcome.

***Case Presentation:*** This was a 47-year-old female patient with a body mass index of 27.34 kg/m^2^ without backache, low back pain, hematuria, urinary urgency, frequent urination, dysuria, and other symptoms. Contrast-enhanced CT scan of the kidney on admission showed four masses in the left kidney and two masses in the right kidney. Preoperative serum creatinine (SCr) was 87 μmol/L. Operation was performed under general anesthesia by first laying the patient in a left lateral position. A 2-cm longitudinal transumbilical skin incision was made to expose the right kidney for complete dissection of the two tumors. First, puncture biopsy was performed, and then two freeze–thaw cryoablation cycles for the two tumors were performed. At last, the patient was laid in a right lateral position for radical nephrectomy of the left kidney. The operative duration, cryoablation time, and estimated blood loss were 200 minutes, 40 minutes, and 100 mL, respectively. Postoperative pathological examination revealed clear-cell renal cell carcinoma. The right glomerular filtration rate tested was 42.36 mL/minute and SCr was 131 μmol/L at day 5 after surgery. There was no evidence of contrast enhancement at the cryoablative region as shown by renal contrasted CT scan performed 4 days after surgery and renal contrasted MRI scan performed 6 weeks after surgery, indicating that there was no tumor remnant or recurrence.

***Conclusion:*** Our preliminary experience shows that the treatment of bilateral renal tumors with unilateral renal tumor cryoablation and contralateral radical nephrectomy by transumbilical 3D LESS is safe, feasible, and effective. It may prove to be a viable option for patients with significant comorbidities and an insensitive treatment intention.

## Introduction

With the development and wide-spread use of imaging technologies in recent years, the detection rate of small renal masses (SRMs) <4 cm in diameter has increased substantially.^1^ At the same time, nephron-sparing surgery (NSS) has been employed in a variety of situations. The general principle of treatment is to resect the tumor effectively, preserve the maximum degree of renal motility, and minimize surgical trauma simultaneously. In 2008, Kaouk et al.^2^ first reported their experience with laparoscopic cryoablation of renal tumors by combining the latest minimally invasive laparoscopic surgery with NSS in neonatal patients with high risk of nephrectomy. However, there is no report about surgical therapy for bilateral renal tumors by using three-dimensional (3D) laparoendoscopic single-site (LESS) surgery in China. After completing all necessary preoperative evaluations and obtaining informed consent from the patients, we carried out the first case of bilateral renal tumor resection through unilateral renal tumor cryoablation and contralateral renal radical resection of bilateral renal carcinoma by transumbilical 3D LESS surgery in China. The aim of this study is to sum up our operational experience in this case, explore the safety and feasibility of the procedure, and evaluate the surgical outcome.

## Presentation of Case

### Materials and Methods

#### Patient's general information

**FIG. 1.**
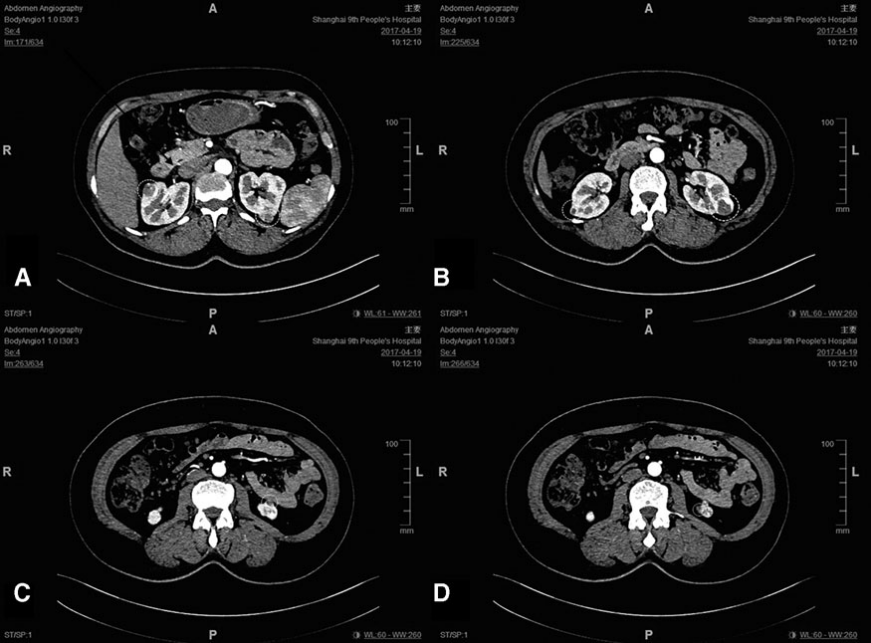
Preoperative contrast-enhanced CT scan of the kidney. **(A)** A mass in the right kidney and a mass in the upper pole of the left kidney. **(B)** A mass in the right kidney and a mass in the middle pole of the left kidney. **(C)** One mass in the lower pole of the left kidney. **(D)** Another mass in the lower pole of the left kidney. The *dotted circles* indicate the tumor before surgery.

This was a 47-year-old female patient with a body mass index of 27.34 (kg/m^2^) and an American Society of Anesthesiologists score of grade I without complaints of backache, low back pain, hematuria, urinary urgency, frequent urination, and dysuria. Physical examination on admission revealed bilateral renal tumors. On April 19, 2017, preoperative enhanced CT scan of the kidney showed four masses in the left kidney and two masses in the right kidney, which were suspected as bilateral renal cancers. The four tumors in the left kidney were located as follows: one in the upper pole, one in the middle pole, and two in the lower pole, with a diameter about 1, 2.5, 0.5, and 0.5 cm, respectively. The two tumors in the right kidney were located one in the upper pole and one in the middle pole, with a diameter of about 1.4 and 0.7 cm, respectively (Fig. 1). Preoperative serum creatinine (SCr) was 87 μmol/L.

#### Medical ethics and informed consent

The study was approved by the Ethics Committee of the Ninth People's Hospital affiliated to Shanghai Jiao Tong University School of Medicine (Shanghai, China), and met the requirements of Helsinki Medical Declaration. The patient was informed of the pros and cons of different surgical procedures in detail, and the possibilities of using an additional auxiliary trocar and surgical transit or diversion to open surgery before signing the informed consent form.

#### Surgical instruments: Multichannel single-site combination kit

TriPort™ single-site laparoscopic surgery channel system (Advanced Surgical Concepts, Wicklow, Ireland). This channel consists of two parts: one is the skin–muscle retractor, including an inner ring and two outer rings, wrapped in double-layer round plastic; the other is the multichannel device, including one 10 mm bushing, two 5 mm bushings, one independent intake passage, and one independent exhaust passage. Endoscopic system: 3D laparoscopic system (Karl Storz, Tuttlingen, Germany). Frozen surgical system: The United States EndoCare company (Cryocare Surgical System) four knife cryopreservation system. The frozen head is a 2-mm diameter right angle head and can be used for direct puncture, with a cold medium argon pressure of 6000PSI and hot medium helium pressure of 2500PSI.

### Right renal tumor cryoablation by 3D LESS

#### Posture and TriPort location

After general anesthesia, the patient was laid on the left side at 70° with the indwelling catheter. The right upper limb was put across the trunk and supported by the bracket and the waist bridge was elevated. All pressure parts of the body were protected by pads, and the legs, buttocks, and shoulders were fixed with wide tapes. To prepare for conversion to open surgery, the disinfection range was the same as traditional open surgery. A 2-cm longitudinal transumbilical skin incision was made to enter the abdominal cavity layer by layer suitable for TriPort placement and maintenance of good air tightness. The Single-Hole Multichannel Combination Kit was placed according the manufacturer's instructions. The ventral machine was connected to a constant pressure of 13 mm Hg (1 mm Hg = 0.133 kPa) to fill the Storz 3D Laparoscopic Kit (Fig. 3A).

#### Dissection of the kidney around the tissue and exposure of the tumor

**FIG. 2.**
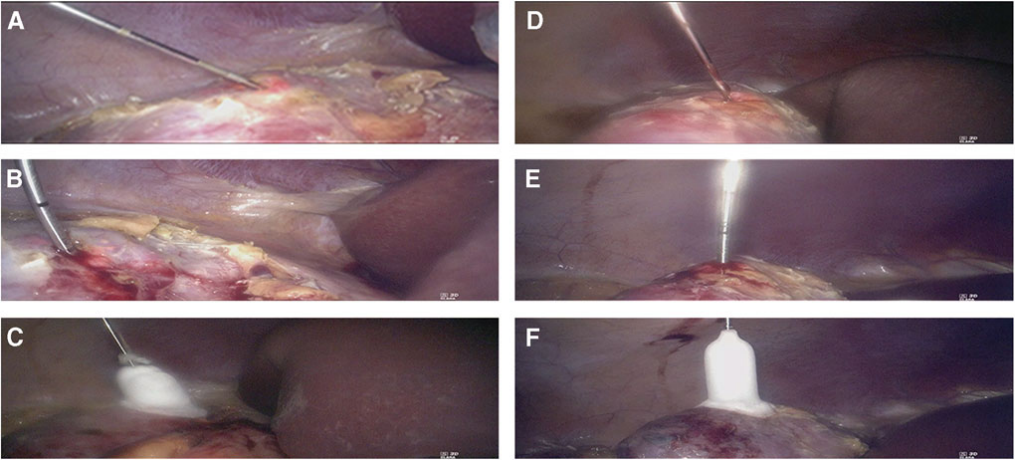
Laparoscopic internal scenes of intraoperative operation. **(A)** Puncture biopsy of the tumor in the upper pole of the right kidney. **(B)** A 2-mm cold knife is put into the tumor in the upper pole of the right kidney. **(C)** The ice hockey is advanced to the upper pole of the kidney and covers the entire tumor. **(D)** Puncture biopsy of the tumor in the middle pole of the right kidney. **(E)** A 2-mm cold knife is put into the tumor body in the middle pole of the right kidney. **(F)** The ice hockey is advanced to the middle pole of the kidney and covers the entire tumor.

The paracolic sulci of the ascending colon was opened with the ultrasound knife to expose the perirenal fascia, free the perirenal fat appropriately, and find the kidney capsule. The surfaces of the two tumors on the right kidney were fully exposed along the renal capsule. The diameter of the two tumors in the upper and middle pole of the right kidney was about 1.4 and 0.7 cm, respectively. Using a puncture needle, puncture biopsy was performed for pathological evaluation (Fig. 2A, D).

#### Cryoablation

**FIG. 3.**
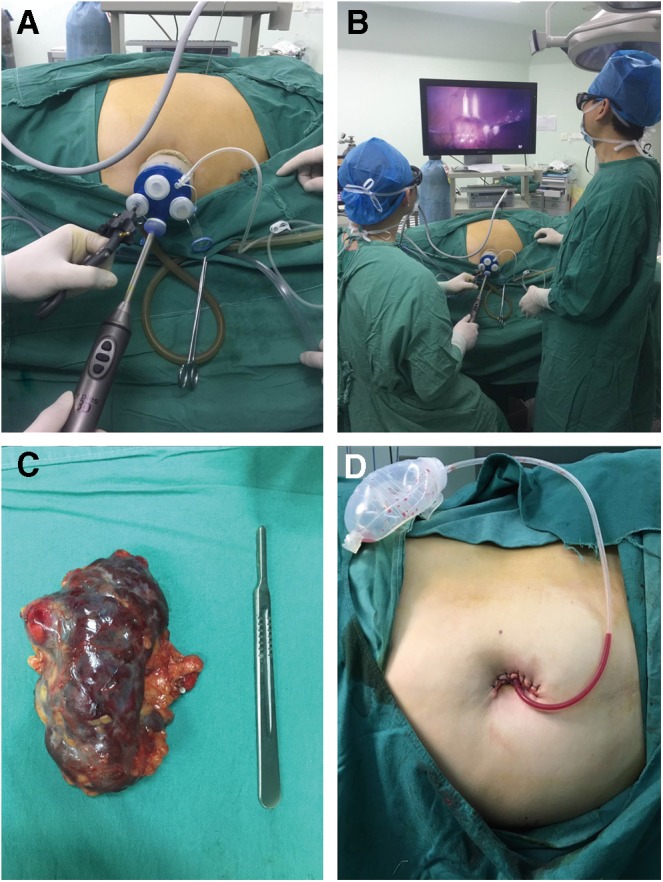
Laparoscopic external scenes of intraoperative operation. **(A)** Port position. **(B)** Intraoperative cryoablation (location). **(C)** A postoperative gross specimen. **(D)** A view of the postoperative incision.

Under laparoscopic direct vision, percutaneous puncture was made. According to the tumor size, location and depth, a 2-mm cold knife was put into the upper pole tumor (Fig. 2B). At <1 cm from the edge of the tumor, the tip of cold knife was advanced close to the tumor capsule. When the tumor was subjected to a −40°C ice hockey area, the temperature of the cold knife tip was dropped from −80°C to −150°C quickly and remained in a frozen state for 10 minutes; the ice hockey was then progressed to cover the entire tumor within the laparoscopic observation (Fig. 2C); after 5-minute active 100% power rewarming, two-cycle freezing and repeated heating, the frozen knife tip was withdrawn. Cryoablation of the tumor in the middle pole of the right kidney was performed in the same way (Fig. 2E, F). The location of cryoablation is shown in Figure 3B.

### Radical nephrectomy of the left kidney by 3D LESS

The patient was then turned to the right-side (70°) lying position, redisinfected and redraped. Radical nephrectomy of the left kidney was performed through the primary umbilical single-site multichannel Trocar. This is important because we have not added any new wounds. After dissecting the omental adhesion, descending colon, and splenic and renal ligaments, the colon was placed to the bottom to expose the perirenal fat capsule; the renal door was separated and the left kidney was freed to find the renal vein near the kidney door level. After freeing the renal vein, the spermatic vein was cut off to find the left renal artery behind the left renal vein. The left renal artery was freed, ligated, and cut off. The renal vein was treated in the same way. The extensive adhesions in the posterior, anterior, and upper poles of the left kidney were dissected. After freeing the lower pole of the left kidney, the left ureter was cut off. The left kidney and ureter were put into the specimen bag and drawn out of the body. After checking for no obvious bleeding, a negative-pressure drain tube was indwelled in the abdominal cavity. The single-site laparoscopic operation Trocar was removed. The peritoneum and the myocutaneous and subcutaneous skin were sewed up (Fig. 3C, D).

## Results

**FIG. 4.**
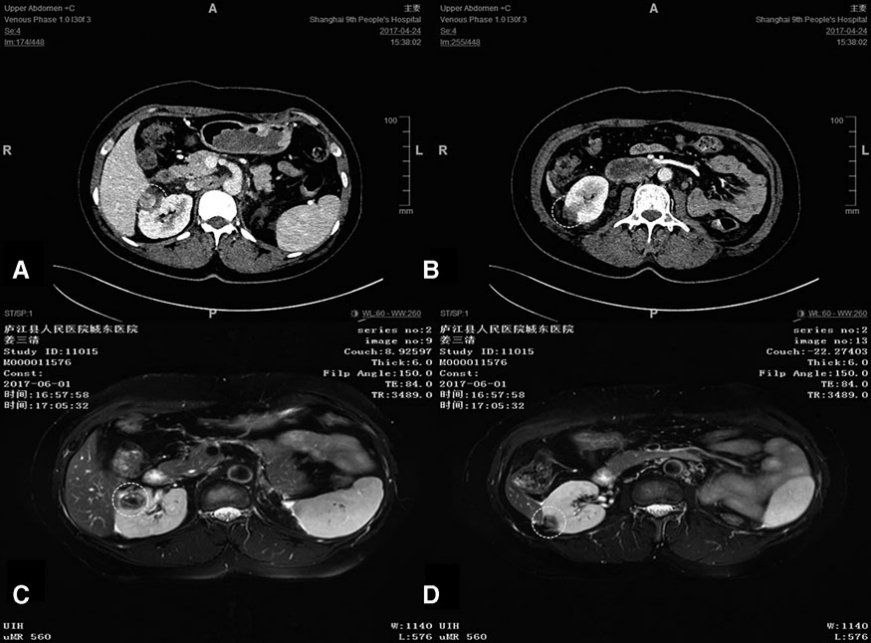
Contrast-enhanced CT scan 4 days after surgery, and contrast-enhanced MRI scan of the kidney 6 weeks after surgery. **(A)** The mass in upper pole of the right kidney 4 days after surgery. **(B)** The mass in the middle pole of the right kidney 4 days after surgery. **(C)** The mass in the upper pole of the right kidney 6 weeks after surgery. **(D)** The mass in the middle pole of the right kidney 6 weeks after surgery. The *dotted circles* indicate the ablation part of the tumor after surgery.

The procedures were completed successfully without using any additional Trocar. The operation time, cryoablation time, and estimated blood loss were 200 minutes, 40 minutes, and 100 mL, respectively. Postoperative pain measured by VASP scale at days 1, 2, and 3 was 1, 1, and 0, respectively. No intra- or postoperative complication occurred. The first day drainage after surgery was 60 mL, and the negative-pressure drainage tube was removed. Histopathological assessment showed that both kidneys were clear-cell carcinoma and the left kidney was classified as Fuhrman grade I. The glomerular filtration rate of the right kidney was 42.36 mL/minute. SCr was 131 μmol/L at day 5 after surgery, and 111.4 μmol/L 2 weeks after surgery. There was no evidence of contrast enhancement in the cryoablative region by renal contrast-enhanced CT scan performed 4 days after surgery and by renal contrast MRI scan performed 6 weeks after surgery, indicating that there was no tumor remnant or recurrence (Fig. 4A, B: 4 days after surgery enhanced CT scan of kidney; Fig. 4C, D: 6 weeks after surgery enhanced MRI scan of kidney).

## Discussion and Literature Review

In 2006, a large-scale case analysis by Huang et al.^3^ showed that radical nephrectomy was a new independent risk factor for postoperative chronic kidney disease, with an occurrence rate as high as 65% 3 years after surgery. Therefore, minimally invasive surgery of ablation is a minimally invasive subgroup that retains the combined advantage of NSS for SRMs in patients with a poor general condition and apprehension of NSS. Cryoablation and radiofrequency ablation are the most widely used and most intensively studied energy ablation techniques. Despite the lack of long-term follow-up data, current studies have shown that laparoscopic cryoablation of renal tumors is one of the most advantageous techniques. On the basis of wealth single-site laparoscopic operation experience, we carried out the first clinical series by retroperitoneal LESS renal cryoablation and the first clinical case by transumbilical LESS renal cryoablation in China. The recent effect is exact. It has obvious advantages especially for elderly patients or patients with more complications. Although the patient had no obvious preoperative complications, she had bilateral renal tumors, two in the right kidney and four in the left kidney. In fact, according to the size of the tumor, bilateral cryoablation is an optional method. However, when evaluating preoperative CT, we found that there were more left renal tumors (four tumors found after surgery). In addition, preoperative CT also found some suspicious satellite stoves, and to prevent the recurrence of tumor postoperatively, after full communication with patients and their families, we chose such a surgical approach. We tried to cryoablate the two tumors in the right kidney and radically resect the left kidney by transumbilical 3D LESS for the first time in China, in an attempt to enrich the surgery approach of LESS for renal tumor cryoablation.

Adequate perioperative preparations are necessary for LESS of renal tumor cryoablation/radical nephrectomy. Retroperitoneal LESS renal cryoablation is usually indicated for tumors close to the posterior kidney. But, the patient had bilateral kidney tumors, although she had no past history of abdominal surgery. Give, the large abdominal space and easiness for tumor exposure, transumbilical LESS was finally chosen for bilateral surgery. A CT-guided percutaneous cryoablation *in vitro* is a good method, but its application has limitations. First of all, it is more suitable for the dorsal mass near the kidney; if the lump is near the ventral side, percutaneous puncture could cause the risk of abdominal organ damage; Second, we performed the entire surgery by a single-hole laparoscopic surgery. Just changing the position of the patient during operation can treat both kidneys simultaneously without additional wounds, and laparoscopic cryoablation is more intuitive and accurate than percutaneous cryoablation.

Successful cryoablation refers to the complete loss of contrast enhancement on follow-up CT or MRI after surgery, indicating complete tissue destruction. It was reported that follow-up observation should be performed by CT or MRI every 3 months within 12–18 months after surgery and then yearly to determine whether the tumor was completely destroyed or the lesion remained in a stable state, and whether there were signs of tumor in the surgical area.^4^ In our patient, contrast-enhanced CT scan performed 4 days after surgery and contrast-enhanced MRI scan performed 6 weeks after surgery showed that the spherical frozen area and edge enhancement disappeared, the necrotic tissue was gradually absorbed, and the tumor size remained stable, indicating that no tumor remnant or recurrence occurred. The short-term effect is exact in our patient. Postoperative follow-up observation showed no significant reduction in renal function as compared with the preoperative condition.

In summary, unilateral renal tumor cryoablation and contralateral radical nephrectomy of bilateral renal tumors by transumbilical 3D LESS surgery is an important extension of the LESS technique in the research of retaining unilateral neonatal surgery of bilateral renal tumors. It has advantages of small trauma, mild postoperative pain, quick recovery, light renal function damage, and the short-term curative effect, and therefore has a good clinical application prospect. However, as there is limited clinical experience with this technique, clinical indications should be observed with caution with respect to long-term renal function and tumor control, and more randomized controlled studies are required before it can be applied as a routine in clinical practice.

## Abbreviations Used

3D: three-dimensional
LESS: laparoendoscopic single-site
NSS: nephron-sparing surgery
SCr: serum creatinine
SRMs: small renal masses

## References

[1] Hollingsworth JM, Miller DC, Daignault S, Hollenbeck BK Rising incidence of small renal masses: A need to reassess treatment effect. J Natl Cancer Inst 2006;98:1331–1334 16985252 10.1093/jnci/djj362

[2] Kaouk JH, Haber GP, Goel RK, et al. Single-port laparoscopic surgery in urology: Initial experience. Urology 2008;71:3–6 18242353 10.1016/j.urology.2007.11.034

[3] Huang WC, Levey AS, Serio AM, et al. Chronic kidney disease after nephrectomy in patients with renal cortical tumours: A retrospective cohort study. Lancet Oncol 2006;7:735–740 16945768 10.1016/S1470-2045(06)70803-8 PMC2239298

[4] Tracy CR, Cadeddu JA Chapter 62. Nonsurgical focal therapy for renal tumors. In: Wein AJ, Kavoussi LR, Partin AW, Peters CA, eds. Campbell-Walsh Urology, 11th ed. Philadelphia, PA: Saunders, 2016, pp. 1484–1499

